# Social Media Addiction during COVID-19-Mandated Physical Distancing: Relatedness Needs as Motives

**DOI:** 10.3390/ijerph19084621

**Published:** 2022-04-12

**Authors:** Cecilia Cheng, Yan-Ching Lau

**Affiliations:** 1 Department of Psychology, The University of Hong Kong, Pokfulam, Hong Kong; 2 Division of Psychology and Language Sciences, University College London, Gower Street, London WC1E 6BT, UK; sophie.lau.18@ucl.ac.uk

**Keywords:** information technology addiction, mental health, psychological need, social distancing, social media use, social networking

## Abstract

In the early stages of the coronavirus disease 2019 (COVID-19) pandemic, national lockdowns and stay-at-home orders were implemented by many countries to curb the rate of infection. An extended stay-at-home period can frustrate people’s need for relatedness, with many turning to social media to interact with others in the outside world. However, social media use may be maladaptive due to its associations with social media addiction and psychosocial problems. Our study was set at this special context to examine the associations among social media addiction, two aspects of relatedness needs (i.e., satisfaction and frustration), and two prominent psychosocial problems (i.e., depressive symptoms and loneliness). The participants were 1048 residents of the U.K. or U.S. (46% men, mean age = 44.10 years, *SD* = 12.59, age range: 18–65). The results indicated significant positive associations between relatedness need frustration and social media addiction as well as significant positive associations between social media addiction and the two types of psychosocial problems. More importantly, both of these significant associations were strong enough to partially explain the significant positive associations between relatedness need frustration and the two types of psychosocial problems. These findings provided some support for relatedness need frustration as a motivation of social media addiction.

## 1. Introduction

Coronavirus disease 2019 (COVID-19) is caused by an unknown highly transmissible virus [1,2]. This novel disease quickly escalated into a global pandemic that has affected more than 325 million people and caused approximately 5.5 million deaths worldwide as of 15 January 2022 [3]. To mitigate the rapid spread of the COVID-19 infection in the pandemic’s early stages, many countries implemented the drastic public health control measures of national lockdowns or stay-at-home orders [4,5,6,7]. Depression and loneliness were the two “signature mental health concerns” during the COVID-19 era [8,9,10,11].

The residents of countries with stay-at-home orders in effect could not maintain contact with others through face-to-face interactions. A recent meta-analysis revealed social isolation to be consistently associated with both mental and physical health problems [12], although most of the studies included in the meta-analysis investigated subjective rather than objective social isolation. The present study extends the literature by examining objective social isolation; actually, such isolation was mandatory due to the implementation of stay-at-home orders during the COVID-19 pandemic.

### 1.1. Prevalence of Social Media Addiction during COVID-19 Pandemic

In the present cyber era, people interact with others in two worlds: the real and the cyber worlds. With face-to-face interactions restricted under a national lockdown or stay-at-home orders, social media emerged as a major means of connecting with social network members [13,14]. A previous study revealed time spent with family members and friends to be inversely associated with internet use [15]. It is not surprising that Facebook, one of the most commonly used social media platforms, has reported a 70% increase in time spent on the platform and more than a 50% increase in messaging since the onset of the pandemic [16]. As the mandated physical distancing measures deprived individuals of opportunities to connect with their social network members in person, many of them turned to social media to maintain existing offline relations via online platforms [17,18,19,20].

The optimal use of social media has been found to foster psychological well-being through the development of new social relations and the strengthening of existing ties [21,22,23]. However, social media use should only be regarded as a supplement to face-to-face interactions rather than a surrogate for social connections in real life or a replacement for a loss of real-life social support [24]. Such a notion stems from a study that compared the levels of life satisfaction among social media, phone, and in-person communication [25]. The findings indicate that highly active users tend to experience a reduction in levels of life satisfaction after using social media, whereas both in-person and phone interactions are positively associated with life satisfaction.

Apart from exerting immediate undesirable effects, excessive use of social media has also been found to incur more long-term maladaptive consequences, with social media addiction as the most common type of such problems. Social media addiction refers to a type of behavioural addiction characterized by an over-concern about using social media and an uncontrollable urge to log on to or use social media [26,27]. Individuals with social media addiction are characterized by an array of symptoms, including a preoccupation with using social media, the development of tolerance symptoms, and a failure to stop using social media despite experiencing adverse consequences [7,28]. Such a devotion of abundant time and effort to social media use has been found to impair functioning in important life domains, especially interpersonal relations [26,29]. In addition, a myriad of studies have documented positive associations between social media addiction and a range of psychosocial problems [30,31,32,33]. Since the onset of the COVID-19 pandemic, social media addiction has been found to be prevalent across countries [28,34,35,36] and across social media platforms [35].

### 1.2. Relatedness Needs as Behavioural Motives and Experiential Requirements

The implementation of the unprecedented stay-at-home orders also disrupted certain fundamental needs, especially those related to social interactions. According to the basic psychological needs theory, one of the subsidiary theories of the self-determination theory [37], the foundation of mental health is the gratification of three basic needs, two of which are personal (needs for autonomy and competence) and the other of which is social (need for relatedness) in nature. Espousing a nuanced view, the two-process model of psychological needs further decomposes basic needs into two elements: need frustration and need satisfaction [38,39]. These two elements of basic needs play distinct roles: needs-as-motives and needs-as-requirements. Specifically, need frustration operates as an underlying motivation that drives and guides individuals to engage in certain behaviours (needs-as-motives), whereas need satisfaction serves as an experiential condition derived from the individuals’ antecedent behaviours (needs-as-requirements).

Of the three fundamental needs highlighted in this theory, relatedness need was considered most likely to influence mental wellness in the context of COVID-19-mandated physical distancing. Relatedness need refers to the desire to be connected to and maintain optimal relations with others [37]. As social animals, human beings have a strong need to belong and affiliate with other people [40]. However, when the stay-at-home orders were in place, the relatedness needs of the residents in the affected regions could not be gratified. As mentioned at the outset, many of the residents turned to social media in an attempt to gain social compensation, and social media addiction was common during the lockdown period in many countries [28,34,35,36]. The constructs of relatedness need frustration and relatedness need satisfaction as well as their respective functions may shed light on individual differences in susceptibility to social media addiction.

#### 1.2.1. Relatedness Needs as Behavioural Motives of Social Media Addiction

From the perspective of the classic theories of motivation [41,42], relatedness need can be viewed as an underlying motivation that drives individuals to engage in certain behaviours and guides their subsequent actions (needs-as-motives). If individuals’ relatedness needs in the real-life social environment are thwarted, they are motivated to seek compensation through interacting with others on social media platforms [43,44]. Empirical data have supported this notion by revealing that social media addiction was positively associated with relatedness need frustration [45]. In addition, previous studies have documented the intervention roles of social media addiction and relatedness need satisfaction in improving mental health problems, particularly depressive symptoms and loneliness [46,47,48,49].

**Figure 1:**
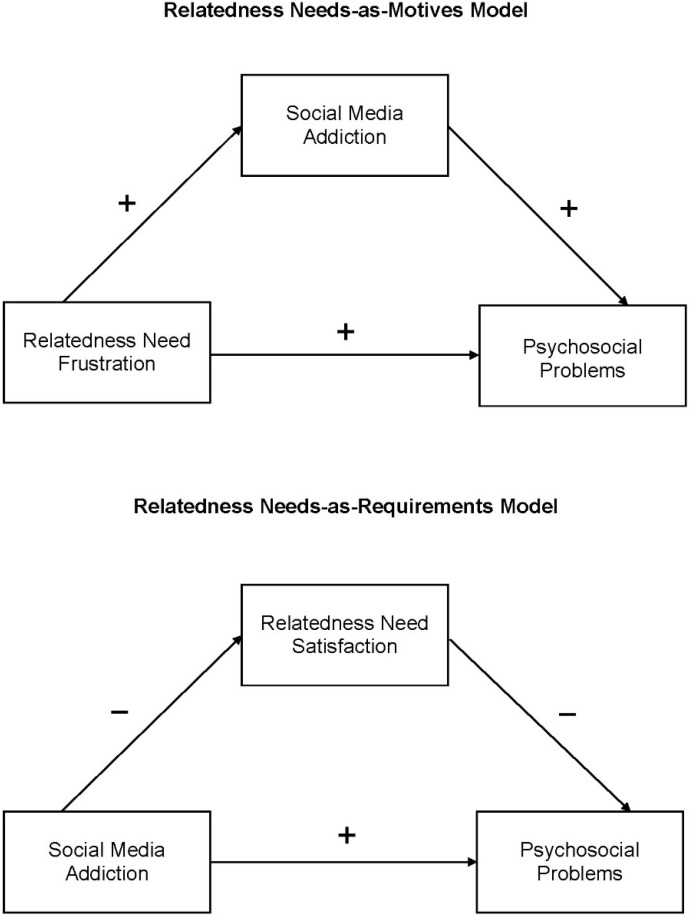
Conceptual frameworks of the relatedness needs-as-motives and the relatedness needs-as-requirements models.

In light of these theoretical postulations and empirical findings, a relatedness needs-as-motives model was formulated, and a set of hypotheses derived from this model is summarized in the upper panel of Figure 1. As similar tendencies of heavy use of social media and social media addiction prevalence were observed during COVID-19-mandated physical distancing, this study was set in this specific context to evaluate the empirical validity of the relatedness needs-as-motives model through testing the following hypotheses:

**Hypothesis** **1** **(H1):** *Relatedness need frustration is positively associated with psychosocial problems (i.e., depressive symptoms and loneliness).*

**Hypothesis** **2** **(H2):** *Relatedness need frustration is positively associated with social media addiction.*

**Hypothesis** **3** **(H3):** *Social media addiction is positively associated with psychosocial problems.*

**Hypothesis** **4** **(H4):** *Social media addiction mediates the positive association between relatedness need frustration and psychosocial problems (relatedness needs-as-motives model).*

#### 1.2.2. Relatedness Needs as Experiential Requirements of Social Media Addiction

For the basic psychological needs theory, a major tenet is that basic needs are “psychological nutrients” that are crucial for psychological adjustment and personal growth [50]. As mentioned above, individuals are motivated to engage in social media to compensate for a perceived loss in the social support that was originally rendered through face-to-face interactions; such attempts of social compensation seeking are largely futile [21]. Similar to other types of behavioural addiction, such as internet gaming addiction [51], social media addiction has been consistently found to be positively associated with problems in romantic and other social relations [52,53], resulting in attachment anxiety, relational ambivalence, and relational dissatisfaction [54,55]. Furthermore, the failure to gratify relatedness needs has been found to be associated with psychosocial problems [56,57]. In light of these theoretical postulations and empirical findings, a relatedness needs-as-requirements model was formulated, and a set of hypotheses derived from this model is shown in the lower panel of Figure 1. Another aim of the present study was to evaluate the empirical validity of this model in the pandemic context through testing the following hypotheses:

**Hypothesis** **5** **(H5):** *Social media addiction is negatively associated with relatedness need satisfaction.*

**Hypothesis** **6** **(H6):** *Relatedness need satisfaction is negatively associated with psychosocial problems.*

**Hypothesis** **7** **(H7):** *Relatedness need satisfaction mediates the positive association between social media addiction and psychosocial problems (relatedness needs-as-requirements model).*

## 2. Materials and Methods

### 2.1. Study Sample

To be eligible for participation in this study, participants needed to be between 18 and 65 years of age, residing in the U.K. or U.S. at the time of the study, able to read English, and willing to give consent. The study sample comprised 1048 adults (46% men, mean age = 44.10 years, *SD* = 12.59, age range: 18–65) recruited from the online crowdsourcing site Prolific Academic. Fifty-five percent of the participants were from the U.K., with the remainder from the U.S. These countries were selected because both were English-speaking countries that were hard hit by the COVID-19 pandemic in its initial stage, and stay-at-home orders were implemented at about the same time of the pandemic. As the samples were heterogeneous in terms of demographic characteristics, with age in particular, the participants were categorized into three age groups: younger adults (aged 18 to 34), middle-aged adults (aged 35 to 49), and older adults (aged 50 to 65).

### 2.2. Measures

The relatedness need satisfaction subscale of the Basic Psychological Need Satisfaction and Frustration Scale [58] was adopted to assess the gratification of the psychological need for relatedness. The subscale consists of four items, such as “I feel connected with people who care for me, and for whom I care”. Each item was rated along a 5-point Likert scale (1 = *not true at all*; 5 = *completely true*). Higher scores indicate greater satisfaction of relatedness needs. The Cronbach’s alpha for the subscale was 0.91 in this study.

The relatedness need frustration subscale of the aforementioned scale was used to examine the frustration derived from the thwarting of this psychological need. The subscale comprises four items (sample item: “I feel excluded from the group I want to belong to”) scored on a 5-point Likert scale ranging from 1 (*not true at all*) to 5 (*completely true*). Higher scores indicate greater frustration of relatedness needs. This subscale was internally consistent, with a Cronbach’s alpha of 0.88 in the present study.

The Bergen Social Media Addiction Scale [59] was employed to measure social media addiction because this is the most popular measure of this type of information technology addiction to date [7]. A sample item of this scale was “[I] felt an urge to use social media more and more”. Each of the measure’s six items was scored on a 5-point Likert scale ranging from 1 (*very rarely*) to 5 (*very often*), with higher scores indicating greater severity of social media addiction. The Cronbach’s alpha was 0.87 in the study.

The Center for Epidemiologic Studies Depression Scale [60] was used to assess depressive symptoms. This scale consisted of 20 items, such as “I felt depressed,” and “I felt hopeful about the future” (inverse scoring). These items were scored on a 4-point Likert scale ranging from 0 (*rarely or none of the time*) to 3 (*most or all of the time*), with higher scores indicating more depressive symptoms experienced during the week prior to the survey. The instrument’s Cronbach’s alpha was 0.91 in this study.

Finally, the UCLA Three-Item Loneliness Scale [61] was adopted to measure loneliness, with items scored on a 4-point Likert scale ranging from 1 (*never*) to 4 (*often*). A sample item was “How often do you feel that you lack companionship?”. Higher scores indicate greater levels of loneliness. The Cronbach’s alpha in the present sample was 0.87.

### 2.3. Procedures

The study protocol was constructed according to the tenets of the Declaration of Helsinki, and the protocol was reviewed and approved by the human research ethics board of the corresponding author’s university prior to survey commencement.

When the participants signed up for the study, all of them were informed of the aim and procedures of the study and were required to provide electronic informed consent before the survey began. The online survey was created and administered through a web browser using Qualtrics^®^ XM (Qualtrics, Provo, UT, USA; http://www.qualtrics.com, accessed on 3 March 2020). Upon survey completion, all of the participants were remunerated according to the payoff scheme recommended by the crowdsourcing site.

### 2.4. Statistical Analyses

Before performing model testing, preliminary analyses were conducted to examine possible differences in the levels of study variables by three major demographic variables, namely nation (UK vs. US), sex, and age (younger adult, middle-aged adult, and older adult). If significant differences were found, two types of post-hoc tests—independent-samples *t*-test (for the dummy variables of nation and sex) and general linear modelling (GLM; for the three-level variable of age group)—would be undertaken. For all the analyses in this study, the Bonferroni correction was applied to minimize the probability of Type I errors arising from multiple comparisons [62].

For testing the two models (see Figure 1), mediation analysis (Model 4) was performed using the PROCESS macro for SPSS (version 3.5) [63]. Each model was tested twice, each with a criterion variable (i.e., depressive symptoms or loneliness). The hypothesized direct and indirect effects were tested using an ordinary least squares path analysis to estimate the coefficients in the process model. The analysis was conducted using the bootstrap method, which did not require the meeting of the assumption of a normal sampling distribution for indirect effects. Following the recommendations by Hayes [64], 5000 bootstrap samples were used to yield 95% bias-corrected and accelerated confidence intervals (CI) for a statistical inference about the indirect effects [64]. An indirect effect was considered statistically significant if its 95% CI did not include zero. Prior to analysis, all of the variable means were centred to reduce the potential for collinearity.

## 3. Results

### 3.1. Descriptive Statistics

Overall, the results from the GLM showed significant differences in the levels of study variables between the sexes and among the three age groups (*p*s < 0.001), but there were no significant differences between the UK and the US samples (*p* = 0.22). No interaction effects among these demographic variables were found (*p*s > 0.18).

For tests of sex differences, female participants were found to generally report higher levels of relatedness need satisfaction, social media addiction, and depressive symptoms than their male counterparts (*p*s < 0.005). However, there were no significant differences in the levels of relatedness need frustration and loneliness between male and female participants (*p*s > 0.57).

For tests of age differences, there were differences among the three age groups in all of the study variables (*p*s < 0.001). The participants in the younger-adult group had lower levels of relatedness need satisfaction but higher levels of loneliness than those in the other two age groups (*p*s < 0.001). Those in the older-adult group reported lower levels of relatedness need frustration and depressive symptoms than their counterparts in the other two age groups (*p*s < 0.04). For social media addiction, the participants in the younger-adult group had higher scores than those in the middle-aged adult group, who in turn had higher scores than those in the older adult group (*p*s < 0.002). Descriptive statistics of these demographic groups are shown in Table 1.

The inter-correlations among the study variables are reported in Table 2. As shown in the table, the hypothesized positive association between relatedness need frustration and social media addiction, those between relatedness need frustration and both psychosocial problems, as well as those between social media addiction and both psychosocial problems were all significant (*p*s < 0.001), providing empirical support for Hypotheses 1, 2, and 3. In addition, the hypothesized negative associations between relatedness need satisfaction and the two psychosocial problems were significant (*p*s < 0.001), providing support for Hypothesis 6. However, the hypothesized negative association between relatedness need satisfaction and social media addiction was marginally significant (*p* = 0.06), and Hypothesis 5 was not supported. The same pattern of findings was replicated using a partial correlation analysis that controlled for the effects of sex and age.

### 3.2. Mediation Analysis for Model Testing

**Figure 2:**
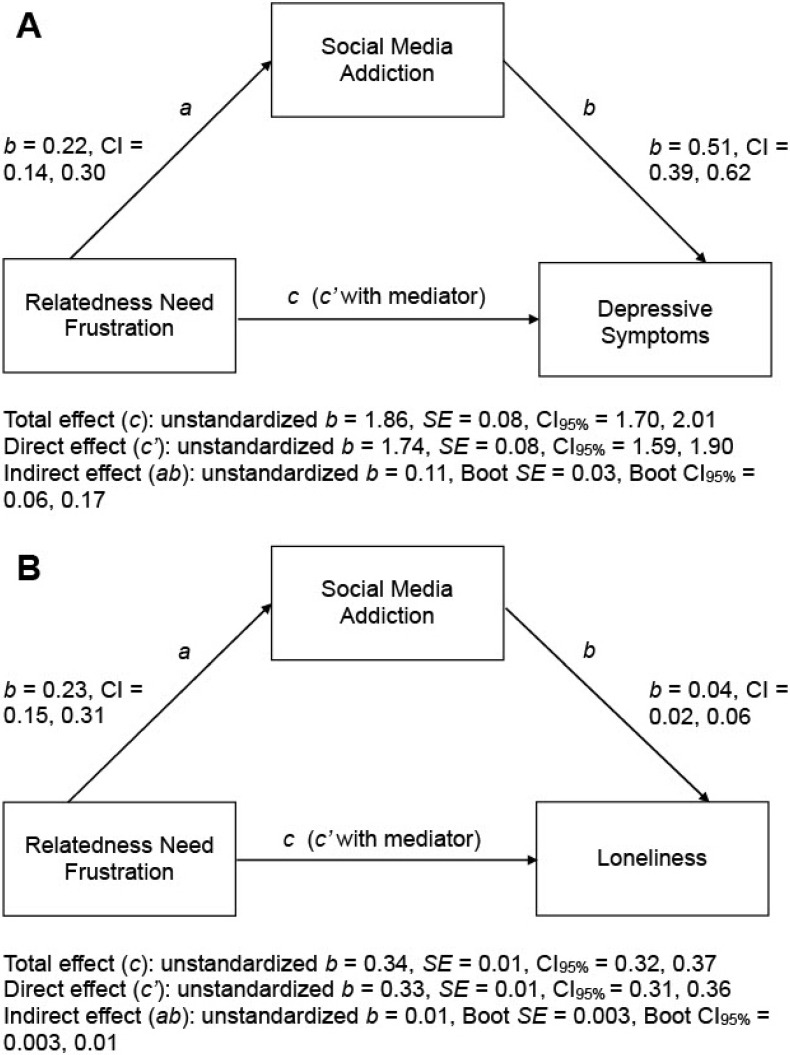
Summary of mediation analyses for testing the relatedness needs-as-motives model with depressive symptoms (**A**) or loneliness (**B**) as the criterion variable (*n* = 1045). Bootstrap analysis of 5000 samples. Sex and age were entered as covariates. All displayed values are unstandardized path coefficients. Boot = bootstrap; *SE* = standard error; CI = confidence interval.

**Figure 3:**
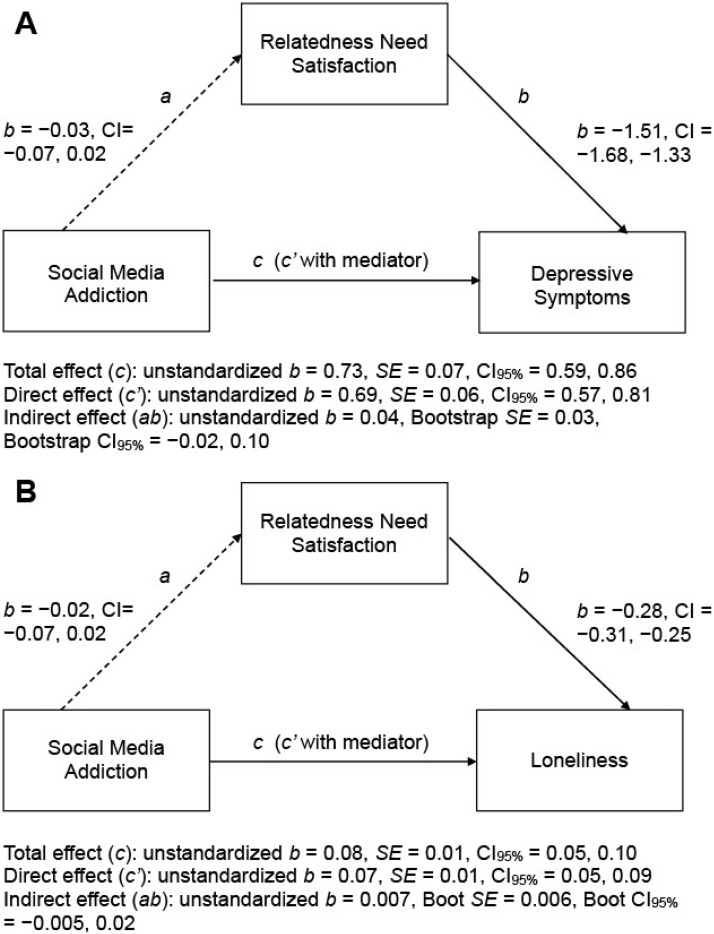
Summary of mediation analyses for testing the relatedness needs-as-requirements model with depressive symptoms (**A**) or loneliness (**B**) as the criterion variable (*n* = 1045). Bootstrap analysis of 5000 samples. Sex and age were entered as covariates. All displayed values are unstandardized path coefficients. Non-significant paths are represented by dashed arrows. Boot = bootstrap; *SE* = standard error; CI = confidence interval.

The results from the mediation analysis are summarized in Figure 2 and Figure 3. The relatedness needs-as-motives model (i.e., Hypothesis 4) was tested first. Conditional on the model assumption of “relatedness need frustration ➔ social media addiction ➔ psychosocial problems”, the mediation analysis showed that the pairwise correlations between relatedness need frustration and social media addiction as well as between social media addiction and depressive symptoms were strong enough to partially explain the positive association between relatedness need frustration and depressive symptoms (see Figure 2A). The same pattern of mediation findings was also found for the criterion of loneliness (see Figure 2B). Taken together, these results were consistent with the predictions of the relatedness needs-as-motives model. 

The relatedness needs-as-requirements model (i.e., Hypothesis 7) was then tested. Conditional on the model assumption of “social media addiction ➔ relatedness need satisfaction ➔ psychosocial problems”, the mediation analysis revealed that only the pairwise correlation between relatedness need satisfaction and depressive symptoms was significant, but that between social media addiction and relatedness need satisfaction was not significant. These two correlations were not strong enough to account for the positive association between social media addiction and depressive symptoms (see Figure 3A). An identical pattern of mediation findings was obtained for the test with loneliness as the criterion variable (see Figure 3B). In summary, these findings were not in line with the predictions of the relatedness needs-as-requirements model.

## 4. Discussion

The foregoing findings examined two hypothesized aspects of relatedness needs (i.e., needs-as-motives and needs-as-requirements) and their associations with the social media addiction and psychosocial problems experienced when the stay-at-home orders were implemented during an early stage of the COVID-19 pandemic. These orders were mandated to reduce human contact for the purpose of public health enhancement or maintenance but have had unintended side effects, including social media addiction and some psychosocial problems associated with relatedness need frustration. During physical isolation, many residents in affected regions became reliant on social media as an attempt to compensate for a loss of the social support that is normally rendered through face-to-face interactions. Unfortunately, social media use can turn into an addiction that is itself associated with depressive symptoms and loneliness.

### 4.1. Relatedness Needs as Behavioural Motives of Social Media Addiction

Adopting a nuanced approach to the study of relatedness need, the present study investigated this fundamental need as both behavioural motives and experiential requirements. The present findings provided some empirical support for the relatedness needs-as-motives model but not the relatedness needs-as-requirements model in the context of COVID-19-mandated physical distancing. In this unusual context, the implementation of such unprecedented, drastic health control measures had elicited considerable psychosocial problems among the residents of the affected regions. According to the basic psychological needs theory, the frustration of psychological needs represents stronger unpleasant experiences and, thus, plays a more influential role in uncontrollable, threatening circumstances, whereas the satisfaction of psychological needs represents stronger fulfilling experiences and, thus, plays a more influential role in volitional, challenging circumstances [50]. Such conceptual distinctions may explain, in part, why only relatedness need frustration was associated with social media addiction and psychosocial problems in the specific context when people had very little to do on their part to interact with others and obtain social support through face-to-face interactions.

Another possible explanation is that need satisfaction reflects more self-determined forms of behavioural regulation, with behaviours driven primarily by internal contingencies [37]. The construct of need satisfaction is, thus, more apt to explain and predict psychological phenomena that bolster optimal daily functioning, personal growth, and mental wellness. In contrast, need frustration reflects the adoption of more controlled forms of behavioural regulations, with behaviours driven by both internal and external contingencies [50]. The construct of need frustration is, thus, more apt to explain and predict psychological phenomena that enhance vulnerabilities for daily dysfunctioning, defensiveness, and psychopathological issues. As shown in this study, relatedness need frustration was positively associated with social media addiction, which is characterized by a lack of self-regulation in social media use that impairs psychological functioning and elicits a range of psychosocial problems [7,65]. These findings, thus, imply the importance of examining need satisfaction and need frustration as two distinct constructs when making behavioural predictions and explanations.

### 4.2. Research and Practical Implications

The escalation in the popularity of various social media platforms has been accompanied by a declining trend in non-digital social interactions among North Americans, young adults in particular [66], and less time interacting with others in person is likely to undermine psychological well-being and social skills while increasing loneliness and depressive symptoms. Although online communication offers such perks as the elimination of time and geographical constraints, users must be wary of the possible maladaptive consequences of over-reliance on technology to fulfil their relatedness needs while physically isolated. The possible consequences include a host of psychological symptoms akin to mental disorders, including an inability to regulate attention, insomnia, stress, compulsive behaviour, separation anxiety, and depression [67,68].

The findings of this study showed that social media addiction had a weak association with relatedness need satisfaction but strong associations with psychosocial problems, thereby providing some evidence of the “village effect” proposed by Pinker [69]. Pinker argues that people with more active in-person social networks tend to have longer lifespans, regardless of their subjective appraisal of loneliness, level of introversion, or both, thus suggesting that face-to-face contact is irreplaceable in the digital era. In-person social contact has been demonstrated to both strengthen the immune system and buffer against mental health decline, psychological and physical benefits that are largely absent in online interactions [69].

Attempts to gratify relatedness needs online are not straightforward. Those who rely mainly on social media to stay connected with their social network members may be vulnerable to social media addiction. One way to mitigate this common type of behavioural addiction is to be mindful of the tendency to check one’s social media accounts throughout the day simply to fill time, a phenomenon that has been commonly observed during the physical distancing periods mandated by the COVID-19 pandemic. The tendency, dubbed “habit checking” [70], can lead to social media addiction. The habitual use of mobile apps contributes to smartphone addiction over time [71]. Users are advised to set a limit on the amount of time they spend on social media per day or to use mobile apps that deliberately block social media access during particular periods of the day, such as while working from home or attending lectures at home during class suspensions.

### 4.3. Study Limitations and Research Directions

Several caveats must be noted before concluding the paper. First, the study focused on the relatedness need, which is only one of the three fundamental psychological needs posited in the basic psychological needs theory. Research has shown that the interaction among the three needs collectively plays an influential role in mental health [72], with each need exerting a direct impact and mediating effect on distinct dimensions of mental health. Hence, future studies should include other psychological needs in the protocols to evaluate how the interaction among various needs influences social media addiction and mental health in the pandemic context.

Second, we asked the participants to self-report core symptoms of social media addiction but did not assess the cognitive appraisals that may have led to such addiction. Accordingly, we cannot specify the cognitive mechanisms associated with psychosocial problems in the social media context. Mackson, et al. [73] reported an association between low self-esteem and heightened anxiety levels among Instagram users as well as higher depression levels resulting from upward social comparison. A fruitful direction for future research would be to focus on cognitive appraisals and mechanisms to provide further insight into the development of social media addiction while coping with physical distancing and ways to prevent this type of addiction during this difficult period.

Third, it is important to reiterate that the samples were drawn from the U.K. or the U.S., both of which are individualist countries with a high level of economic development. Previous multinational research indicates vast cultural differences in levels of various types of information technology addiction and mental wellness [74,75,76]. As the COVID-19 pandemic has swept the globe and affected the subjective well-being of myriads of residents worldwide [77,78,79,80,81,82], more collaborative effort should be taken among scholars from different cultural regions for identifying cultural difference and invariance in the behaviour and emotional reactions among people from the diverse countries that have been hit hard by COVID-19.

Finally, as we collected data from a single time point during the pandemic in this study, it should be noted that the present results cannot be taken to imply causal links, and it would, thus, be inappropriate to conclude that relatedness need frustration causes social media addiction and psychosocial problems. As previous research conducted amid the SARS epidemic showed considerable variations in public responses across various waves [83], follow-up research is recommended to examine psychosocial changes over time. For example, a follow-up study would be helpful to determine whether any of the individuals who did not report symptoms of social media addition at an initial time point develop such symptoms over time and how increased social media use for a prolonged period of physical distancing elicits greater maladaptive psychosocial outcomes. Such longitudinal data would be highly valuable for informing the design of prevention programs aimed at early intervention to alleviate social media addiction and mental health issues [84].

## 5. Conclusions

The pandemic is expected to continue to disrupt everyday life for many people all over the world, at least until effective vaccines are available for mass immunization. Quarantine mandates are, thus, likely to continue to be implemented and relaxed as cases of COVID-19 infection wax and wane, thereby necessitating varying periods of physical distancing. Following the postulations of the two-process model of psychological needs, the study distinguished between relatedness need frustration and relatedness need satisfaction and found that social media addiction was related to the former construct but not the latter. In line with the predictions of the relatedness needs-as-motives model, the findings of this study indicate that frustration of the fundamental psychological need for relatedness when human contact is minimized is associated with both social media addiction and psychosocial problems. These findings highlight a possible trade-off between physical and mental health during the pandemic: physical distancing can limit COVID-19 infection and transmission, albeit at the expense of compromised digital and mental wellness. Hence, in addition to the implementation of public health control measures to curb viral transmission, public health organizations should also impose effective measures to bolster both digital well-being and mental health.

## Figures and Tables

**Table 1:** Descriptive statistics of study variables by sex and age groups.

		Male (*n* = 481)		Female (*n* = 564)	
Study Variable	Adult Group	Mean	*SD*	Mean	*SD*
Relatedness need frustration	younger	7.98	3.42	7.65	3.70
	middle-aged	7.28	3.41	7.04	3.50
	older	6.29	2.76	6.74	3.56
Relatedness need satisfaction	younger	15.11	3.30	16.67	3.04
	middle-aged	16.59	3.07	17.01	3.18
	older	17.01	2.81	17.13	3.52
Social media addiction	younger	10.60	4.88	13.51	5.53
	middle-aged	10.13	4.55	11.54	5.13
	older	8.18	3.39	9.84	3.96
Depressive symptoms	younger	17.27	10.46	20.38	11.99
	middle-aged	16.78	10.48	17.36	12.06
	older	12.83	9.69	16.21	10.59
Loneliness	younger	5.09	1.86	5.33	2.02
	middle-aged	5.00	1.86	4.74	1.87
	older	4.56	1.86	4.76	1.89

*Note.* Three participants did not report their sex.

**Table 2:** Zero-order Pearson correlations among the study variables (*n* = 1048).

Study Variable	2	3	4	5
1. Relatedness need frustration	−0.70 **	0.20 **	0.60 **	0.63 **
2. Relatedness need satisfaction	--	−0.06	−0.46 **	−0.50 **
3. Social media addiction		--	0.35 **	0.21 **
4. Depressive symptoms			--	0.67 **
5. Loneliness				--

** *p* < 0.001 (Bonferroni correction).

## Data Availability

The data presented in this study are available on request from the corresponding author.
